# Enhancing Objective Structured Clinical Examinations through visualisation of checklist scores and global rating scale

**DOI:** 10.5116/ijme.5ad4.509b

**Published:** 2018-05-04

**Authors:** Mohsen Tavakol, Gill Pinner

**Affiliations:** 1 The University of Nottingham, Medical Education Center, Nottingham, UK

**Keywords:** Objective Structured Clinical Examinations, visualisation of checklist scores, global rating scale

## Introduction

The effective use of Objective Structured Clinical Examinations (OSCEs) requires careful attention to the construction of clinical scenarios and to the way clinical performance will be measured.

Assessments must be aligned to the curriculum and when constructing an OSCE station, care must be taken to set a scenario which tests the desired learning outcomes.  This allows the student to demonstrate evidence of the clinical skills they have acquired and will create measures of clinical performance likely to be valid and reliable. However, there are many sources of variability which can influence the generalizability and reliability of the scores. For example, the assessors’ judgment, students’ ability and the sampling of tasks/cases.

An assessor’s judgment may lose validity if the tasks within a station are so complex and extensive, resulting in examiner ‘demoralization and fatigue’.^1^ Such errors will distort the measurement and threaten the validity and reliability of the examination. If an OSCE is planned prudently with ‘a common conceptualization’ of performance among assessors when they are observing and rating students,^2^ performance ratings will accurately reflect the clinical skills of students.

The judgments that assessors make may also be prone to measurement error leading to issues of inter-rater reliability i.e., would the student have achieved the same performance rating in the “abdominal” station if a different examiner had rated them?

Performance ratings are susceptible to the various types of internal and external errors which can contribute to less reliable OSCE performance ratings. An example of an internal error would be variations in the level of student’s interest and motivation, whereas differences in circuits, student gender, the ethnicity of standardised patients and students,^3^ sites and examiners are considered as external error factors.^4^ Since the subjectivity in ratings can be a major source of error in OSCEs, one solution to minimise such error and improve OSCE-rating reliability is to obtain ratings from multiple assessors in each station (i.e., each student is rated by two or more independently judging assessors). However, to improve the reliability of an OSCE it is better to increase the number of stations with one examiner present than have fewer stations with two examiners assessing^5^ and is more feasible in practice. In addition, using multiple assessors may not affect systematic errors (for example, a combination of lenient and stringent examiners). Psychometric methods such as Generalizability Theory and Item Response Theory enable us to identify, isolate and estimate sources of measurement error, which has been extensively covered elsewhere.^6^

To standard set an OSCE it is common place to use a borderline group or regression method. This requires assessors to both score the task set in a station, using either a checklist or domain-based marking scheme and also to give their global rating of the overall students’ performance.  It is particularly important that the assessors are aware of the standard of the set of students being examined, especially that of a “borderline candidate”. More than a century ago, Thorndike and Hagan concluded that “the ideal rater is the person who has had a great deal of opportunity to observe the person being rated in situations in which he would be likely to show the qualities on which ratings are desired”.^7^  The discrepancy between assessor checklist scores and their global ratings is likely to have an impact on borderline pass/fail decisions and become a significant source of measurement error.  Studies have raised concerns about common errors in judging student performance ratings.^8^^,^^9^ Indeed, placing students correctly into a specific category (e.g., fail, borderline, good and excellent) can be an indicator of rating accuracy and is necessary for providing effective observational feedback, a key area to address for assessment leads.^2^

### Aim

To our knowledge scant attention has been paid to visualise the checklist scores and global ratings together to obtain a clear picture of the quality of performance ratings. This paper therefore considers assessor performance and addresses how visualising checklist scores and global ratings with boxplots provide substantial detail of the distribution of scores from different assessors examining the same station and its relationship with global ratings.

### Assessor performance 

Assessor bias error occurs when the ratings given are not grounded in the performance of the student on the domain being assessed. For each OSCE station, the performance of the task is rated by assessors who assign a score to each of the items or domains being assessed, creating a total mark achieved by the student.  For standard setting or psychometric study purposes, assessors are also asked to make an overall judgment about the quality of student performance using a global rating scale independent of the individual marks awarded. Many sources of variability can influence such ratings and judgements, resulting in a lack of generalizability and poor reliability of OSCE scores.^10^ Assessor judgments may be prone to generosity error, severity error, central tendency error, halo error (rating based on general impression), contrast effect (examiner ratings is on the basis of the suitability of promptly earlier students) and other unconscious biases. Such errors will distort the measurement and will also be a serious threat to the validity and reliability of OSCEs. In addition, cognitive complexity, another source of variability, can affect the accuracy of judgment ratings. Cognitive complexity refers to “the degree to which a person possesses the ability to perceive behaviour in a multidimensional manner”,^11^ and it is supported by the Cognitive Compatibility Theory.^12^

Experimental studies by Schneier showed that the cognitively complex assessors were more confident in their ratings, were less lenient and subject to less halo effect in their ratings, and also showed less restrictions on range errors. Therefore, it is important for assessment leads to match the cognitive characteristics of assessors to checklists and global rating scales to reduce the bias of leniency and stringency in ratings.  A further external factor which results in a positively or negatively biased assessment is the mood of assessors. According to the Affect Infusion Model, affective states can cause a ‘colouring of the judgemental outcome’.^13^ For example, in performance assessment, assessors in good moods remember more positive information from their own memories, leading to a more positive performance assessment.^14^  A literature review shows that examiners with a positive affect are associated with giving higher ratings, having a larger halo effect and reduced accuracy of performance rating.^15^ These findings show that the psychosocial context of assessors can have an important role in the rating process of OSCE stations.

### Results of assessor ratings

The characteristics of assessors, internal and external error factors, may result in bias of underrating or overrating of student performance.  This in turn threatens the validity and reliability of OSCE scores. One should question the reliability of student scores and the overall fairness of an assessment when there are significant differences between assessor ratings. This may need to be addressed through moderation of the assessment, where marks may be adjusted to compensate for unacceptable levels of variation and error from assessors and other sources; stations be reviewed and improved for future use; and assessment leads consider and implement ways to improve assessor performance.

**Figure 1:**
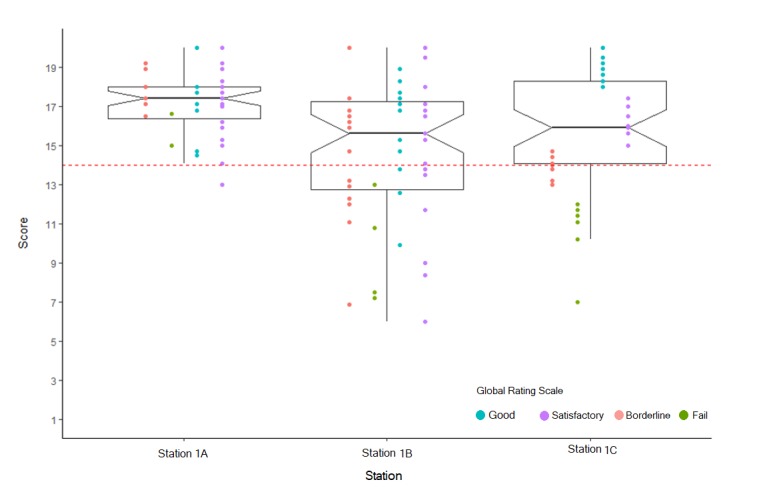
The association between global ratings and checklist scores

There are a number of psychometric methods to estimate such differences between assessors and we will describe one simple yet effective approach. A visual example of this is shown in Figure 1 which illustrates the distribution of scores and global rating (GR) scale from three different assessors examining the same skills station (station 1) across three circuits (A, B, C) of an OSCE. Visualising checklist scores and global ratings with box plots provide substantial detailed information that is easily accessible.

### Checklist scores

Figure 1suggests the total marks awarded from the assessor on Station 1A are more homogenous than for the other two circuits. The notch in Station 1A is not overlapping with notches in other stations suggesting a significant difference between medians across stations.  With only one student failing the station 1A, this may be due to generosity error. Station 1B has a wide range of scores, the most number of fails but also some perfect scores. The notch in Station 1C overlaps with the notch in Station 1B, suggesting that there is no statistically significant difference in medians between Station 1B and Station 1C.

### Global ratings

Figure 1 also provides clear evidence that, in Station1A, two students were not considered to have met the required level of competency overall and rated as “Fail.” However, the scores achieved from the checklist exceeded the cut score, so they actually passed the station. Some students who were rated as “Borderline” had scores overlapping with those rated as “Satisfactory” and even “Good. Similarly, in Station 1B, some students were rated as “Good” by the assessor but failed the station. These stations show the discrepancy between global ratings and checklist scores. On inspecting Station 1C, GRs do not overlap and match the checklist scores demonstrating good assessor alignment. Simple visual presentations such as these plots can be used to improve examiner performance by feeding back the results to assessors so that they can reflect on areas where they may need to recalibrate their expectations of the required competency standard for clinical tasks.

### Leniency and stringency effects

There are different plausible approaches (e.g., Generalizability Theory or Many-Facet Rasch model) to estimate the assessor leniency and stringency effects, to calculate test-score

**Figure 2:**
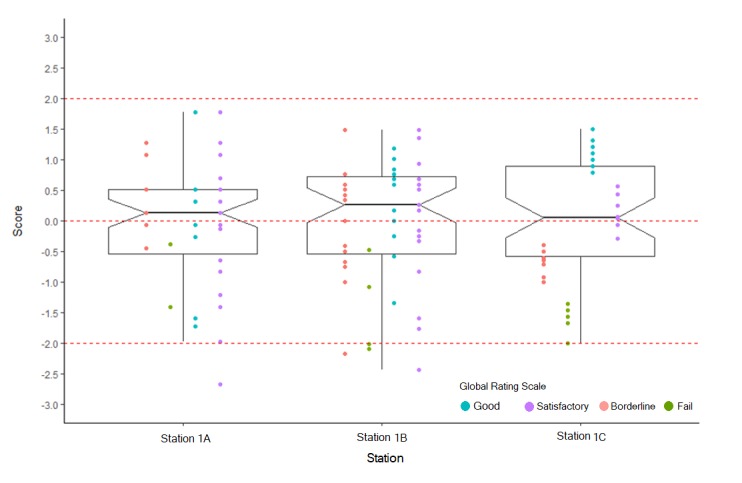
The distribution of assessor’s standard scores

reliability, and to make suggestions for enhancing the rating process,^16^ mainly when each student is assessed by two or more independent assessors. As stated before, in practice it is often not feasible to use two or more independent assessors within each station as it is a major resource issue. Therefore, other methods are required to identify the lenient and stringent assessors by comparing them with each other. Comparing assessors with each other can be done by calculating a statistic called, a standard score or z score. The standard score describes the score relative to the mean in terms of the standard deviation. Fundamentally, the standard score shows how many standard deviations the score is from the mean of a particular distribution. If assessors’ scores are converted to standard scores, we are then able to compare them with each other and see which assessor is lenient or harsh. It is worth noting that standard scores can be positive (higher than the mean) or negative (lower than the mean). In addition, converting scores to standard scores results in a distribution of standard scores with a mean of 0 and a standard deviation of 1.

The larger the standard score, the more extreme the score comparatively to others. Therefore, a standard score of 0.5, being close to the mean, indicates that the assessor was not particularly lenient or harsh. If a standard score is -2 on the whole distribution, this indicates the assessor scored -2 standard deviations below the mean and that the assessor is likely to be stringent or ‘hawkish’ compared to the average assessor. If a standard score is greater than 2 this indicates the assessor is lenient or “dovish”.  Using a standard score greater than 2 or less than -2 is an arbitrary approach. However, when the scores are normally distributed, it is exceptional to get a standard score greater than +3 or less than -3.  When we convert the assessors’ scores to standard scores, we can plot box plots for the standard scores.  As shown in Figure 2, few scores are appeared below -2 standard score, and no score appeared above 2 standard score. This suggests that there is little “dove and hawk” effect on students’ scores using two standard deviations above or below the mean. In addition, the assessors provided a wide range of standard scores.  This indicates that they were using the full range of the rating scales, which may suggest that they were more likely assessing the true behaviour of students based on their performances, but this can only be an assumption. Advanced psychometrics methods, for example, the many-facet Rasch measurement model, are required to detect erratic assessors. If substantial discrepancies are identified, these assessors should undergo further training. Finally, establishing reliable and valid OSCE scores will help identify students who are able to progress, and this may result in better performance when they encounter real clinical situations.

## Conclusions

Improving the quality of assessor observational ratings in OSCEs is very important and this can be achieved through visualisation of checklist scores and global rating scale. As discussed earlier, multiple facets can engender assessor errors and biases, e.g., halo effect, severity or kindness error, central tendency, liking, first impression, companionship, and hence make students’ scores less decisive and unreliable.  However, caution should be taken when interpreting the potential effect of error, as a score may also be a reflection of true performance, and not be affected by potential error.

Box plots are a powerful and useful tool for displaying specific summary statistics in order to obtain an immediate impression of assessor bias effects. What is relevant here is the means by which the box plots can be fed back to assessors so that their ability to discriminate between high and low performers is enhanced. Therefore, based on these boxplots, assessor training on rating accuracy can be discussed and improved so that they can make more informed judgments concerning performance rating of students and hence provide a fairer and more accurate score. Providing accurate scores may also increase the motivation of students, and hence they may consider the feedback provided to enhance their performance.^2^ However, it should be emphasised that in the training of assessors, other criteria should be considered in order to increase rating accuracy (e.g., a common conceptualisation of the domain being assessed or the expected levels of student performance).  A further application of these boxplots is that assessment leads may moderate/adjust OSCE scores to account for ‘dovish’ or ‘hawkish’ assessors. Presenting information to assessors and moderation panels in this simple visual way is more accessible and may have more impact than the presentation of results from advanced psychometric procedures, such as the IRT models which can be complicated to understand.

### Conflict of Interest

The authors declare that they have no conflict of interest.
